# Evaluation of the Association of Plasma Pentraxin 3 Levels with Type 2 Diabetes and Diabetic Nephropathy in a Malay Population

**DOI:** 10.1155/2013/298019

**Published:** 2013-11-20

**Authors:** Norhashimah Abu Seman, Anna Witasp, Wan Nazaimoon Wan Mohamud, Björn Anderstam, Kerstin Brismar, Peter Stenvinkel, Harvest F. Gu

**Affiliations:** ^1^Rolf Luft Research Center for Diabetes and Endocrinology, Department of Molecular Medicine and Surgery, Karolinska University Hospital, Solna, Karolinska Institutet, SE-17176 Stockholm, Sweden; ^2^Cardiovascular, Diabetes and Nutrition Research Centre, Institute for Medical Research, Jalan Pahang, 50588 Kuala Lumpur, Malaysia; ^3^Center for Molecular Medicine, Karolinska Institutet, SE-17176 Stockholm, Sweden; ^4^Division of Renal Medicine, Department of Clinical Science, Intervention and Technology, Karolinska University Hospital, Huddinge, Karolinska Institutet, SE-14157 Stockholm, Sweden

## Abstract

Recent reports have demonstrated that elevated plasma long pentraxin 3 (PTX3) levels are associated with cardiovascular and chronic kidney diseases. In the current study, we investigated the plasma PTX3 levels in 296 Malay subjects including the subjects with normal glucose tolerance (NGT) and type 2 diabetes (T2DM) patients with or without DN by using an enzyme-linked immune-sorbent assay. Results showed that in males, plasma PTX3 levels in T2DM patients without DN were lower than that in the subjects with NGT (2.78 versus 3.98 ng/mL; *P* = 0.021). Plasma PTX3 levels in T2DM patients with DN were decreased compared to the patients without DN (1.63 versus 2.78 ng/mL; *P* = 0.013). In females, however, no significant alteration of plasma PTX3 levels among NGT subjects and T2DM patients with and without DN was detected. Furthermore, an inverse correlation between PTX3 and body mass index was found in male subjects with NGT (*P* = 0.012; *r* = −0.390), but not in male T2DM patients, neither in all females. The current study provided the first evidence that decreased plasma PTX3 levels are associated with T2DM and DN in Malay men and also suggested that PTX3 may have different effects in DN and chronic kidney diseases.

## 1. Introduction

Type 2 diabetes (T2DM) and obesity have become epidemic in Malaysia. According to the latest National Health and Morbidity Surveys, 14.9% of Malaysian adults aged 30 years and above are diabetic and they are often obese or overweight [1, 2]. Moreover, diabetic nephropathy (DN) is the most common cause of end-stage renal disease (ESRD) and contributes to 57% of patients with T2DM in this country. Although T2DM represents a preventable and treatable cause of ESRD, the number of ESRD cases caused by T2DM has increased and accounts for more than 50% of incident dialysis patients [3–5]. The public burden from diabetes and DN is enormous.

Pentraxin 3 (PTX3) is an acute-phase glycoprotein and a soluble receptor acting as an opsonin. PTX3 protein is expressed in vascular endothelial cells and macrophages. Thereby, its levels may reflect more directly the inflammatory status of the vasculature [6, 7]. Recently, several clinical investigations have demonstrated that elevated plasma PTX3 levels are associated with cardiovascular [8, 9] and chronic kidney diseases (CKD) [10, 11]. Furthermore, plasma PTX3 levels are inversely associated with body mass index (BMI) suggesting that PTX3 may play a role in obesity and metabolic syndrome [12, 13]. Interestingly, with the approach of genome-wide scan and linkage analysis, chromosome 3q is found to be linked with diabetes and DN in many ethnic groups [14–16]. The gene encoding for PTX3 protein is located in chromosome 3q25.32 and resides in the linkage region. Yilmaz et al. have shown that PTX3 is positively associated with proteinuria in Turkish subjects with T2DM hypertensive patients, while renin angiotensin system blockade lowers plasma PTX3 levels in the patients [17, 18]. However, there are gender and racial differences of plasma PTX3 levels [7, 19], by which the association of PTX3 with kidney dysfunction may be influenced.

 In the present study, we examined plasma PTX3 levels in a Malay cohort, including NGT subjects, T2DM patients with and without DN. We also analyzed plasma PTX3 levels according to BMI. The aim of our study was to investigate the association of plasma PTX3 levels with T2DM and DN in this Malay population. Data from our study are also useful for better understanding the different effects of PTX3 in DN and CKD. 

## 2. Patients and Methods 

### 2.1. Patients and Controls

Malaysia is a country with multicultures and multiethnic populations. We collected the samples of subjects with NGT and patients with T2DM from the collaborating centers all over Malaysia. The ethnic distribution of our study subjects was 67.6% Malay, 15.3% Indian, 14.8% Chinese, and 2.3% Indigenous Sabahans and Sarawakians. To avoid the error caused by ethnic stratification, Indian, Chinese, Indigenous Sabahans, and Sarawakians were excluded in the present study. Finally, 103 (50 males/53 females) Malay individuals with NGT (controls) and 193 (99/94) Malay patients with T2DM (cases) were included into the analyses. Diagnoses of T2DM were done based on the World Health Organization (WHO) criteria [20]. The diagnoses of DN were based on urine albumin-to-creatinine ratio (ACR) suggested by ADA [21]. The patients with T2DM and normoalbuminuria (ACR < 3.5 mg/mmol) were considered as controls for DN, while the patients with macroalbuminuria (ACR ≥ 35 mg/mmol) and ESRD who needed dialysis were included as the cases for DN. Except for the patients with ESRD, all other subjects were required to give urine under fasting conditions early in the morning. Clinical characteristics of all Malay subjects with NGT and T2DM with and without DN are summarized in Table 1.

All subjects answered a set of questionnaires and underwent clinical and physical examinations. Informed consent was obtained from all subjects, and the study was approved by the local ethical committees. Data and materials transfer agreement from the Institute for Medical Research, Malaysia to Karolinska Institutet, Sweden was signed prior to the study.

### 2.2. Clinical Characterization

Body weight and height were measured using a calibrated digital scale (Seca, Birmingham, UK). The WHO/International Association for the Study of Obesity (IASO)/International Obesity Task Force (IOTF) has proposed BMI cut-off values of 23.0–24.9 kg/m^2^ for classification of overweight and of ≥25.0 kg/m^2^ for obesity for adult Asians [22]. Based on the Malaysian clinical practice guidelines, subjects with BMI value ≥23.0 kg/m^2^ are considered as overweight [23]. Systolic and diastolic blood pressures were measured using a digital sphygmomanometer (Omron Healthcare, Inc., Lake Forest, USA) after 5 minutes resting. Creatinine in serum and urine were measured using Randox Assayed Multisera (Randox Laboratories Ltd., Crumlin, UK). Urine or serum was mixed with sodium hydroxide in biochemistry analyzer (Selectra E). Creatinine in alkaline solution reacted with picric acid to form a coloured complex. The amount of the complex formed was measured at wavelength of 490 to 510 nm. The reading of complex measured is directly proportional to the creatinine concentration. Estimate of glomerular filtration rate (GFR) in the dialysis patient was calculated by the mean of renal urea and creatinine clearance from a 24-hour urine correction [24]. 

### 2.3. Plasma PTX3 Measurement

A total of 25 mL venous blood samples were collected from each subject early in the morning after an overnight fasting and then stored at −80°C. Plasma PTX3 concentrations were determined using a commercial enzyme-linked immunosorbent assay kit (Quantakine DPTX 30; R&D Systems Inc., Minneapolis, USA). Experiments with the PTX3 assay were carried out according to the manufacturer's instructions. Briefly, 20 *μ*L of standard and plasma samples was assayed duplicate in the microtiter plate wells coated with a specific PTX3 monoclonal antibody followed by incubation at room temperature for 2 hours. The wells were then washed four times with a buffered surfactant solution. Anti-PTX3 polyclonal antibody conjugated to alkaline phosphatase was added to each well and incubated for two hours at room temperature. After washing step, 200 *μ*L of substrate solution was added to each well followed by incubation for 30 minutes at room temperature. The solution of 2 N sulfuric acid was added to each well to stop the reaction. Absorbance was measured at 450 nm with corrections set at 540 nm using microplate reader. The values of plasma PTX3 levels were extrapolated from a curve drawn using a standard PTX3. 

### 2.4. Statistical Analyses

All data were expressed as mean (95% CI) for normally distributed variables and as geometric means (95% CI) for nonnormally distributed variables. The Kolmogorov-Smirnov test was initially used to test the data for normality. Normal probability plots were created and parameter distributions were transformed to the common logarithm for obtaining a normal distribution before performing statistical analysis. The one-way analysis of variance (ANOVA) was used for comparisons involving more than two groups or independent *t*-test for comparison between two groups. Pearson and Spearman analyses were conducted to determine correlations with continuous and noncontinuous variables, respectively. Statistical significance was defined as the *P* value of below 0.05. All analyses were performed using PASW Statistic Base 18 (SPSSInc, Chicago, USA).

## 3. Results

### 3.1. Gender Differences of Plasma PTX3 Levels in Malay Subjects

In both males and females, there was no difference in age, waist circumference, and HbA1c between T2DM patients and NGT subjects. Although plasma PTX3 levels in males and females differed significantly, PTX3 had no relationship with age in males (*P* = 0.647) or females (*P* = 0.626). Thus, all subsequent analyses of plasma PTX3 levels were done separately in males and females. Our analyses indicated that plasma PTX3 levels in T2DM patients with and without DN were lower as compared with NGT subjects in males (2.62 versus 3.98 ng/mL; *P* = 0.021) but not in females (3.24 versus 3.09 ng/mL; *P* = 0.748). 

### 3.2. Association of Plasma PTX3 Levels with Type 2 Diabetes and Diabetic Nephropathy

**Figure 1:**
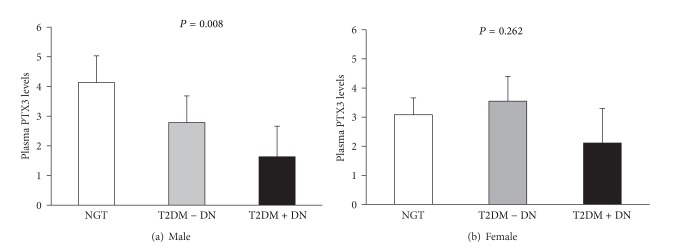
Plasma PTX3 levels in Malay subjects with normal glucose tolerance and type 2 diabetes patients with or without diabetic nephropathy. Data presented as means with 95% CI; *P* values were from ANOVA tests; NGT: normal glucose tolerance; T2DM: type 2 diabetes; DN: diabetic nephropathy.

We further analyzed the association of PTX3 with T2DM and DN.  Figure 1(a) showed that plasma PTX3 levels were consistently decreased from NGT to T2DM without DN and to the patients with DN in males (3.98, 2.78, and 1.63 ng/mL; *P* = 0.008 ANOVA test). Among males, the patients with DN had lower PTX3 levels compared to T2DM without DN (1.63 versus 3.08 ng/mL; *P* = 0.013). In females, however, there was no statistically significant difference of the mean values of plasma PTX3 levels among NGT and T2DM with and without DN (3.09, 3.55, and 2.11 ng/mL; *P* = 0.262, ANOVA test) (Figure 1(b)). 

### 3.3. Correlations of Plasma PTX3 Levels with BMI

**Figure 2:**
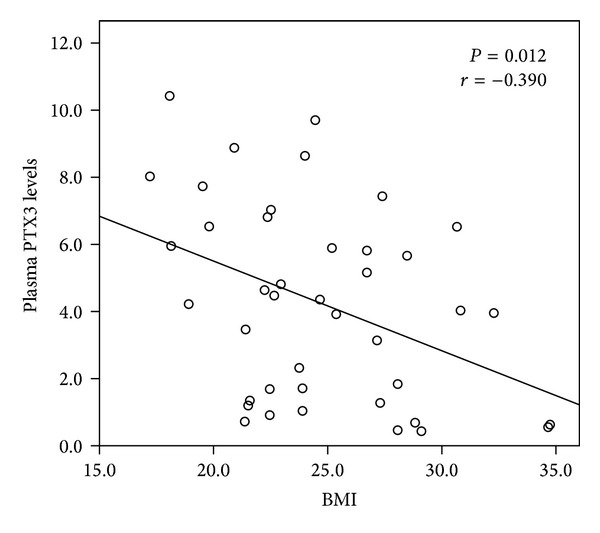
Univariate correlation between plasma PTX3 levels and BMI in Malay men with normal glucose tolerance.

**Figure 3:**
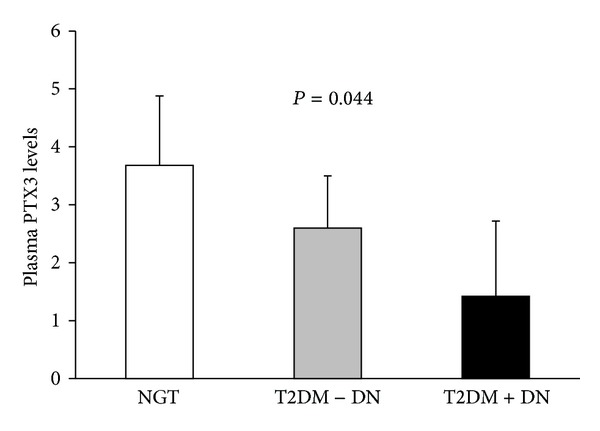
Plasma PTX3 levels in overweight Malay men with normal glucose tolerance and type 2 diabetes patients with or without diabetic nephropathy. Data presented as means with 95% CI; *P* values were from ANOVA tests; NGT: normal glucose tolerance; T2DM: type 2 diabetes; DN: diabetic nephropathy.

There was a negative correlation between plasma PTX3 levels and BMI in male subjects with NGT (*r* = −0.390; *P* = 0.012) (Figure 2) but not in females (*P* = 0.330). The correlation between PTX3 and BMI was not found in all male and female T2DM patients with and without DN. In Malaysia, the adults with BMI value ≥ 23.0 kg/m^2^ are considered to be overweight [23]. To further understand whether the association between PTX3 and DN in T2DM was influenced by BMI, we performed the comparative analyses in the patients with overweight (BMI ≥ 23 kg/m^2^) and lean patients (BMI < 23 kg/m^2^), respectively. In males with overweight, we found that plasma PTX3 levels were gradually decreased from subjects with NGT to T2DM patients without DN and to the patients with DN (3.68, 2.60 and 1.42 ng/mL; *P* = 0.044, ANOVA test) (Figure 3). In lean males and also in all females, plasma PTX3 levels of NGT and T2DM with and without DN were varied but not with any statistical significance.

## 4. Discussion

In the present study, we analyzed plasma PTX3 levels in Malay subjects with NGT and T2DM with and without DN. We report a gender difference of plasma PTX3 levels in this population. Lower levels of PTX3 were found to be associated with T2DM and DN in males but not in females. Furthermore, PTX3 was found to be inversely associated with BMI in males with NGT. The correlation was not observed both in males and females with T2DM and DN.

Gender is an important factor for the development of T2DM and DN. Males have a higher prevalence of T2DM and DN in many populations including Malaysians [1, 3–5]. Epidemiologic reports have demonstrated that DN is 30% more frequent in males than in females [25]. Genetic studies have also showed that DNA polymorphisms in the genes of sex-determining region Y-box 2, angiotensin II type 1, and type 2 receptors are associated with DN with gender-specific effects [26–28]. Previously, Yamasaki et al. observed that plasma PTX3 levels between males and females in a healthy Japanese population are different [7]. In the present study, we demonstrate that plasma PTX3 levels gradually decreased from NGT to T2DM without DN to T2DM with DN particularly among males but not in females. Furthermore, an inverse correlation between PTX3 and BMI was found in male subjects with NGT. This correlation was not seen in all females and males with T2DM and DN. Taking together, data from previous and present studies implicate that PTX3 most likely has gender-specific effects in T2DM and DN, which should be taken into our consideration in further investigations. 

Several studies have reported that increased PTX3 levels are associated with impaired renal function in CKD [10, 11]. The similar association of PTX3 with DN is seen in Turkish patients with T2DM [17]. In the present study, however, we demonstrate that decreased PTX3 levels are associated with DN in Malay men with T2DM. First, the controversy may be caused by the studies in different ethnic populations. Dubin et al. have demonstrated that there are racial differences of PTX3 in term of association with kidney dysfunction [19]. Second, the ages of Turkish T2DM subjects with DN (at 42 years old) [17] are younger and their duration of diabetes are shorter compared to Malay T2DM patients with DN (at the age of 55 years old) in the present study. Furthermore, clinical observations have indicated that two main causes of CKD are diabetes and high blood pressure, which are responsible for up to two-thirds of the cases. The progresses and mechanisms of DN and CKD may be different [29], while PTX3 may have different effects in these two diseases.

We have shown that plasma PTX3 levels were inversely correlated with BMI in males with NGT, which is consistent with previous reports [7, 12, 13]. In the present study, there is a limitation with lack of lean subjects because all patients with T2DM and female subjects with NGT had mean values of BMI at least 27.2 kg/m^2^. Recently, a study has demonstrated that PTX3 is expressed in adipose tissue, and its tissue specific expression reflects endothelial dysfunction [30]. Another study has reported that PTX3 is positively correlated with adiponectin [12]. Although we did not analyze plasma levels of adiponectin in this Malay cohort, the accumulated documents have shown that adiponectin is inversely proportional to obesity and T2DM in different populations including Malaysians. Plasma/serum adiponectin levels in the patients with T2DM and obese subjects are decreased compared to that in healthy control subjects [31, 32]. Therefore, we hypothesize that PTX3, as similar to adiponectin, may have protective effects in increased body weight. Further investigation is needed to fully understand the cellular mechanism of PTX3 reduction in T2DM and DN.

In conclusion, the present study provides the first evidence that decreased plasma PTX3 levels are associated with T2DM and DN in Malay men and also suggests that PTX3 may have different effects in DN and CKD.

## Figures and Tables

**Table 1:** Clinical and laboratory characteristics of Malay subjects with normal glucose tolerance, and type 2 diabetes patients with and without diabetic nephropathy.

Gender	Male				Female			
Group (*N*)	NGT (50)	T2DM − DN (40)	T2DM + DN (59)	*P* values^a/b^	NGT (53)	T2DM − DN (51)	T2DM + DN (43)	*P* values^a/b^
Age (years)	58 (56–60)	55 (53–58)	55 (52–58)		56 (54–59)	53 (50–56)	56 (53–59)	
Diabetic duration (years)	—	14 (12–17)	14 (12–17)		—	12 (9–15)	12 (9–15)	
BMI (kg/m^2^)	24.9 (23.6–26.2)	27.7 (26.4–29.1)	27.6 (26.0–29.1)	0.010/NS	27.2 (25.9–28.5)	29.1 (27.2–30.7)	27.2 (25.1–29.4)	
SBP (mm Hg)	138 (132–144)	137 (129–144)	159 (147–170)	NS/0.001	137 (132–143)	144 (137–150)	149 (137–160)	
DBP (mm Hg)	83 (79–85)	82 (79–85)	83 (79–88)		84 (80–87)	87 (83–91)	79 (73–85)	NS/0.047
FPG (mmol/L)	5.6 (5.0–6.1)	7.3 (5.3–9.4)			5.0 (4.74–5.6)	7.7 (5.6–9.9)		
HbA1c (mmol/mol)	31 (29–33)	43 (33–52)	31 (21–41)		31 (30–33)	38 (30–44)	37 (26–46)	
eGFR (mL/min/1.73 m^2^)	102 (88–117)	93 (81–105)	50 (42–58)	NS/<0.001	103 (92–113)	89 (79–98)	50 (36–64)	NS/<0.001
Creatinine (*µ*mol/L)*	75.9 (69.2–83.2)	81.3 (67.6–93.3)	158.5 (134.9–195.0)	NS/<0.001	57.5 (53.7–63.1)	63.1 (60.3–75.9)	141.3 (112.2–117.8)	NS/<0.001
ACR (mg/mmol)*	0.89 (0.66–1.13)	1.03 (0.63–1.44)	179.0 (133.8–224.3)	0.001/<0.001	0.79 (0.64–0.95)	2.01 (0.25–3.76)	304.3 (149.4–459.3)	0.001/<0.001

Data are expressed as mean (95% CI) for normally distributed variables and as geometric means (95% CI) for nonnormally distributed variables*; NGT: normal glucose tolerance; T2DM: type 2 diabetes; BMI: body mass index; SBP and DBP: systolic and diastolic blood pressures; FPG: Fasting plasma glucose; HbA1c: glycosylated hemoglobin; eGFR: estimated glomerular filtration rate; ACR: albumin creatinine ratio; PTX3: pentraxin 3; *P* values were from tests of NGT versus T2DM − DN^a^ and T2DM − DN versus T2DM + DN^b^.

## Abbreviations

ACR: Urine albumin-to-creatinine ratio
eGFR: Estimate of glomerular filtration rate
DN: Diabetic nephropathy
NGT: Normal glucose tolerance
T2DM: Type 2 diabetes
PTX3: Pentraxin 3.
